# The Role of Heat Shock Protein (Hsp) Chaperones in Environmental Stress Adaptation and Virulence of Plant Pathogenic Bacteria

**DOI:** 10.3390/ijms26020528

**Published:** 2025-01-09

**Authors:** Donata Figaj

**Affiliations:** Department of General and Medical Biochemistry, Faculty of Biology, University of Gdansk, Wita Stwosza 59, 80-308 Gdansk, Poland; donata.figaj@ug.edu.pl or donata.figaj@biol.ug.edu.pl

**Keywords:** heat shock protein (Hsp), chaperones, protein quality control system, plant pathogenic bacteria, virulence, stress response

## Abstract

Plant pathogenic bacteria are responsible for a substantial number of plant diseases worldwide, resulting in significant economic losses. Bacteria are exposed to numerous stress factors during their epiphytic life and within the host. Their ability to survive in the host and cause symptomatic infections depends on their capacity to overcome stressors. Bacteria have evolved a range of defensive and adaptive mechanisms to thrive under varying environmental conditions. One such mechanism involves the induction of chaperone proteins that belong to the heat shock protein (Hsp) family. Together with proteases, these proteins are integral components of the protein quality control system (PQCS), which is essential for maintaining cellular proteostasis. However, knowledge of their action is considerably less extensive than that of human and animal pathogens. This study discusses the modulation of Hsp levels by phytopathogenic bacteria in response to stress conditions, including elevated temperature, oxidative stress, changes in pH or osmolarity of the environment, and variable host conditions during infection. All these factors influence bacterial virulence. Finally, the secretion of GroEL and DnaK proteins outside the bacterial cell is considered a potentially important virulence trait.

## 1. Introduction

Phytopathogenic bacteria, viruses, fungi, and nematodes are responsible for significant losses during the cultivation and storage of agricultural crops, with an estimated annual global cost of USD 220 billion [1]. Bacterial diseases can affect various plant parts, including the roots, stems, leaves, fruits, and tubers [2]. Mitigation strategies for phytopathogenic bacteria include appropriate storage techniques and pathogen control under field conditions. These strategies include chemical pesticides, breeding for more resistant cultivars, and the promising but still developing use of biocontrol agents, such as bacteriophages or beneficial bacteria that promote plant health and growth [3,4,5]. Despite these measures, yield losses still reach significant levels of up to 40% [1]. 

**Figure 1:**
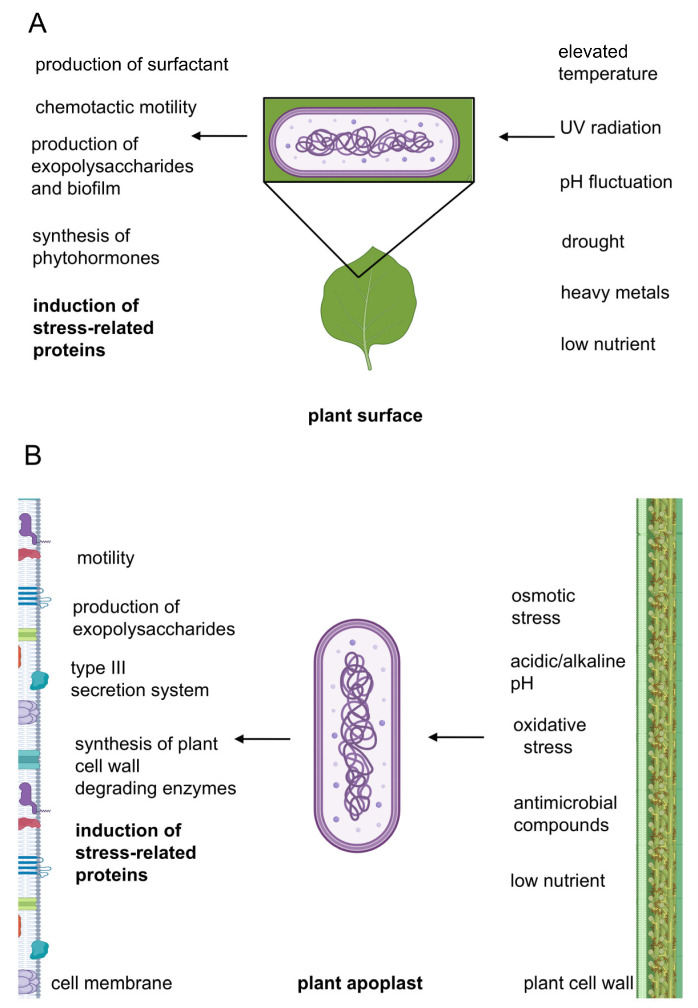
Adaptation of phytopathogenic bacteria to environmental conditions: The right-hand side outlines the stress conditions that bacteria may encounter at different stages of their life cycle, including the epiphytic phase on plant surfaces (**A**) and host infection within the plant, as represented by the interior of the apoplast (**B**). The left-hand side highlights the factors used or produced by bacteria to counteract these unfavorable conditions and induce symptomatic plant infection.

The presence of bacteria and a susceptible plant variety alone is not enough to cause symptomatic infection. Favorable environmental conditions, such as adequate temperature, humidity, and poor oxygen availability, are also required. These three elements collectively form a triangular relationship that defines disease development [6]. Before entering the host, bacteria encounter numerous abiotic stressors during their epiphytic phases of life (Figure 1A). They must survive exposure to high temperatures, UV radiation, drought, heavy metals, fluctuations in soil pH, agricultural antimicrobial compounds, and low nutrient availability. Bacteria employ a range of species-specific strategies to enhance cell survival in response to adverse environmental conditions. These strategies include the production of biofilms and exopolysaccharides (EPSs), which form physical barriers against environmental factors; surfactants that increase the wettability of plant surfaces; chemotactic motility toward more hydrated and nutrient-rich regions; and the production of plant hormones that modulate host behavior [7]. Furthermore, exposure to stressors leads to altered proteostasis at the cellular level. Stress-denatured proteins can form aggregates, and their accumulation may lead to cell death. Consequently, bacteria induce the synthesis of stress-related proteins to protect their proteomes from stress-induced disorders [8]. Under favorable environmental conditions, bacteria enter plant tissues through mechanical tissue damage or open stomatal pores. During colonization of the host, they encounter biotic stress in the apoplast or xylem vessels and face the defense mechanisms of the plant (Figure 1B). After entering the host tissue, bacteria activate defense and adaptive systems that determine their ability to colonize the host and determine whether the infection will progress to the symptomatic phase. The plant environment is predominantly oxygen-limited and acidic. The pH of the apoplast and xylem varies depending on the plant species and tissue type; for example, the pH of the apoplast in maize leaves is approximately 4.9 [9], while the pH of the xylem sap of tomato stems is around 5.25 [10]. Both the apoplast and xylem are nutrient-poor; therefore, bacteria have limited access to essential nutrients, including iron. In response to bacterial invasion, plants initiate the production of free radicals (oxidative burst) [11] and antimicrobial compounds. During plant colonization, the pH of the microenvironment increases. In the later stages of infection, the bacterial species that degrade the cell wall cause tissue maceration, resulting in increased osmolarity. The evolution and adaptation of phytopathogenic bacteria to their host species have led to the development of unique virulence traits that influence the pathogenicity of each bacterium. However, the most common virulence-related traits among phytopathogenic bacteria can be clearly distinguished: (1) the type II secretion system (T2SS), which enables the transfer of pectinases and cellulases into the extracellular space; (2) the type III secretion system (T3SS), which facilitates the secretion of effector molecules into plant cells to modulate plant responses; (3) motility that enables host colonization; (4) production of exopolysaccharides that maintain biofilm integrity; and (5) synthesis of phytotoxins and molecules mimicking plant hormones that modulate plant physiology. A more detailed account of these processes can be found in review papers by Reverchon [6,12] and Melotto [13]. Furthermore, bacteria increase the synthesis of stress-related proteins [8].

To the best of our knowledge, this review represents the first comprehensive examination of the role of chaperone proteins belonging to the heat shock protein family, both in virulence and adaptation to stress conditions, solely in selected species of phytopathogenic bacteria.

## 2. Bacterial Heat Shock Proteins

Heat shock proteins are evolutionarily conserved across all kingdoms of organisms. They function as chaperone proteins that, together with proteases, are integral components of the protein quality control system (PQCS). The optimal functioning of this system is crucial for maintaining proteostasis under both physiological and stress conditions. Initially identified as inducible by heat shock, Hsp chaperones have since been shown to be modulated by other stress factors, including osmotic stress, oxidative stress, changes in pH (both acidic and alkaline), ethanol exposure, and heavy metals. This group of chaperone proteins includes several types that differ in mass and mechanism of action: Hsp100, Hsp90, Hsp70, Hsp60, Hsp40, and small Hsp (sHsp). Under physiological conditions, these chaperones ensure correct protein folding as part of housekeeping activities. In response to stressors, they prevent the aggregation of denatured proteins, disaggregate these aggregates when possible, and facilitate the refolding of the client proteins. The irreversibly damaged proteins that cannot be refolded are directed to the proteolytic pathway. Furthermore, Hsp chaperones perform various functions in prokaryotic and eukaryotic organisms [14]. In pathogenic bacteria, chaperones can serve as virulence factors that enhance the efficiency of the infection process. In humans and animals, perturbations in Hsp levels are associated with tumorigenesis, as well as neurodegenerative, immunological, and cardiovascular diseases [15].

**Figure 2:**
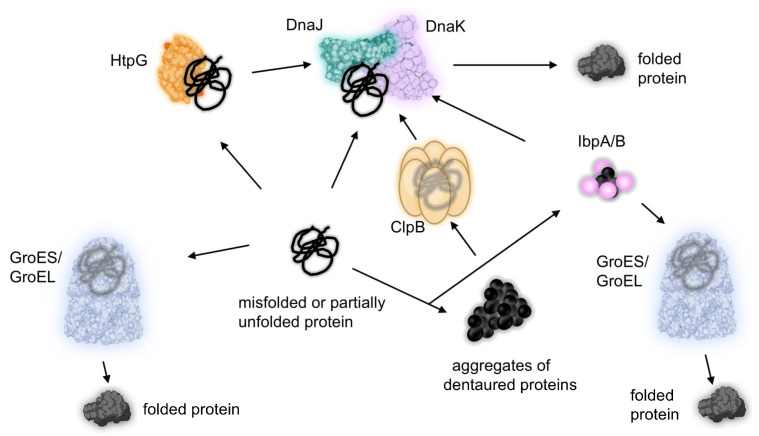
Simplified scheme of Hsp proteins action in *E. coli*. GroEL-GroES and DnaK-DnaJ are the main systems responsible for protein folding. In addition, HtpG cooperates with DnaK in the remodeling of misfolded proteins. ClpB is a disaggregase that interacts with DnaK. IbpA/B proteins function as holdases and cooperate with other chaperones.

Prokaryotic Hsp have been extensively characterized in the model bacterium *Escherichia coli*, where their transcription is regulated by the alternative sigma factor RpoH (sigma32) [16]. These proteins are localized in the cytoplasm, and their function largely depends on ATP hydrolysis. Two major chaperone systems are present, GroEL-GroES (Hsp60-Hsp10) and DnaK-DnaJ-GrpE (Hsp70-Hsp40-nuceotide exchange factor). In addition to these systems, other chaperones, including ClpB (Hsp100), HtpG (Hsp90), and small Hsp (sHsp), IbpA, and IbpB, can cooperate to enhance cellular stress responses (Figure 2).

GroEL (also known as Cpn60) is the sole chaperonin that is indispensable for bacterial viability, with certain exceptions. For example, some bacteria in the Mollicutes class (bacteria lacking the call wall) do not possess this chaperone [17,18]. Notably, it interacts in vivo with only approximately 10% of *E. coli* proteins [19]. Together with its co-chaperonin GroES, GroEL ranks among the 21 most abundant proteins in *E. coli* under physiological conditions, excluding ribosomal proteins [20]. Its oligomeric structure is barrel-shaped and consists of two heptameric rings stacked back-to-back. GroEL binds to non-native client proteins, which are typically misfolded or partially unfolded in the presence of ATP. The heptameric ring-shaped co-chaperonin GroES encapsulates the central cavity of GroEL, creating an environment that is conducive to substrate folding. This folding process is facilitated by ATP hydrolysis. Once folding is complete, GroES dissociates from GroEL, enabling the release of properly folded client proteins [21,22].

In the DnaK-DnaJ-GrpE system, DnaJ binds to a misfolded or partially unfolded client protein and transfers it to DnaK. The folding of this substrate by DnaK occurs simultaneously with the hydrolysis of ATP to ADP. The dissociation of ADP and rebinding of ATP, mediated by GrpE, acts as a signal for the release of properly folded substrate from DnaK. While DnaK can function independently, cooperation among these three proteins is essential for optimal folding efficiency [23,24]. Furthermore, DnaJ can function independently of other chaperones, exhibiting an aggregation-suppressing effect [24,25].

ClpB, a ring-shaped hexamer, exhibits disaggregase activity by facilitating the release of denatured proteins from the aggregates. The release of denatured proteins from these aggregates and the unfolding of their polypeptide chains within the oligomeric structure of ClpB depend on ATP hydrolysis. Although ClpB can independently reactivate some substrates, its efficiency in protein refolding is significantly enhanced when it cooperates with the DnaK chaperone system [26]. Initially, DnaJ associates with these aggregates and recruits DnaK, which subsequently forms an ATP-dependent complex with ClpB [27,28].

The Hsp90 HtpG protein primarily functions in the folding and remodeling of non-native and unfolded proteins, in collaboration with DnaK [29]. The substrate is transferred to DnaK through an asymmetric mechanism in which one monomer of DnaK interacts with the HtpG dimer [30]. ATP hydrolysis to ADP enables the folding of the client proteins. Subsequently, the remodeled protein is released as a result of ADP dissociation [31]. In the absence of DnaK, HtpG can act as a holdase in an ATP-independent manner, thereby preventing aggregation of client proteins [32].

IbpA and IbpB sHsp are ATP-independent chaperones that function as holdases to prevent aggregation of denatured or unfolded proteins. Because they lack foldase activity, once cellular homeostasis is restored, client proteins are transferred to other chaperone systems for reactivation [33]. Although IbpA and IbpB exhibit distinct functions, the presence of both is necessary for the optimal functioning of the ClpB-DnaK system [34]. Small Hsp chaperones interact to form heterodimers, which can then reorganize into higher-order oligomeric structures [35].

### 2.1. Heat Shock Proteins of Plant Pathogenic Bacteria

The present study focuses on a selected group of phytopathogenic bacteria that are considered among the ten most significant from an economic and scientific perspective [2]. A concise overview of the characteristics of these bacteria, including their hosts, disease symptoms, and representative set of virulence traits, is presented in Table 1.

To date, the molecular chaperone function of Hsp homologs in phytopathogenic bacteria has been empirically validated in three species.

DnaK from *A. tumefaciens* acts as a molecular chaperone both in vivo and in vitro. It complements deletions and mutations in the *dnaK* gene of *E. coli*, restoring bacterial growth at non-permissive temperatures. Moreover, it displays basal ATPase activity, which increases 2-fold in the presence of *Agrobacterium* DnaJ protein [57]. Furthermore, unlike DnaK from *E. coli*, it effectively prevents the aggregation of thermally denatured malate dehydrogenase [58].

The four small heat shock proteins from *A. tumefaciens*, HspL, HspC, HspTA1, and HspTA2, protect citrate synthase from thermal aggregation in vitro. This protective effect depends on the formation of large oligomeric structures. Additionally, HspL and HspAT2 protect VirB8, a protein associated with type IV secretion system (T4SS) assembly in *A. tumefaciens*, from thermal-induced aggregation. Among these proteins, only HspL exhibits optimal efficiency, suggesting that it serves as a chaperone for VirB8 [59].

Another protein from *Agrobacterium*, DnaJ, facilitates the growth of *E. coli* lacking the functional *dnaJ* gene [57]. When considered alongside the in vitro data presented by Hennessy [57], this provides substantial evidence for its function as a molecular chaperone.

The *dnaK* gene in *P. syringae* pv. *syringae* complements the *dnaK* mutation in *E. coli* in vivo, confirming the molecular chaperone function of *Pseudomonas* DnaK.

HspA, an sHsp from *X. campestris*, protects *E. coli* proteins, partially protects firefly luciferase against heat-induced aggregation, and can reactivate heat-denatured EcoRI enzyme [60].

#### 2.1.1. Heat Shock Proteins Levels Are Regulated in Response to In Vitro Stress Factors

In *A. tumefaciens,* the transcriptional regulation of *hspL* is RpoH-dependent, whereas *hspAT1* and *hspAT2* are post-transcriptionally regulated through ROSE, a motif characteristic of rhizobial small heat shock genes. This sequence is localized in the 5′ untranslated region and adopts a secondary structure at lower temperatures, which obscures the Shine–Dalgarno sequence. The expression of *hspL*, *hspAT1*, and *hspAT2* is induced by heat shock. A temperature shift from 25 °C to 37 °C resulted in a greater than 10-fold increase in transcript levels of *hspL* and a 5-fold increase in *hspAT1*. HspC is not heat-induced [61]. HspL synthesis increases in the presence of acetosyringone (AS), an inducer of virulence *vir* genes [62]. This protein induction is indirectly dependent on the expression of the *virB* gene, which is essential for T4SS assembly, T-DNA transfer to plant cells, and tumor formation in the host organism [63]. The *groEL* and *groES* genes are transcribed as polycistronic mRNAs [64], and the *groE* operon is regulated by RpoH and HrcA (protein acting as a transcriptional repressor under physiological conditions) [65]. Under heat shock conditions at 42 °C, a cleavage event occurs between the *groEL* and *groES* genes in the mRNA, resulting in the production of *groEL* as the dominant monocistronic mRNA, while *groES* becomes unstable and readily degraded. This phenomenon may represent an additional regulatory mechanism for this operon at higher temperatures [64]. The expression of *groEL* and *dnaK* was induced by elevated temperatures and the presence of ethanol. The transcription of *dnaK* and *groEL* increased by approximately 5- and 4-fold, respectively, in the presence of 4% ethanol [66]. Exposure of bacteria to 42 °C resulted in significantly higher expression levels of *dnaK* and *groEL*, although this increase exhibited distinct dynamics. The transcriptional peak for *groEL* occurred at 5 min, as reported by Segal [67] for the *groE* operon, demonstrating an upregulation of approximately 3.5-fold. Maximum gene expression for *dnaK* was observed at the 20 min time point, with a 35-fold increase [66]. Transcriptomic and proteomic data are in concordance, as the levels of DnaK and GroEL proteins in the cell are elevated after temperature shifts to 39 °C or 45 °C [68]. Furthermore, exposure of cells to 37 °C, a moderate stress factor for plant bacteria, also results in increased synthesis of GroEL, DnaK, and ClpB [65]. The induction of GroEL at 42 °C was also confirmed by Rosen et al. [69]. Moreover, elevated levels of GroES were observed under these conditions, which was consistent with the transcriptomic data. Conversely, the levels of GroEL and GroES remained unaltered under oxidative stress induced by 2 mM hydrogen peroxide. However, an analysis of *groEL* gene expression demonstrated a decrease over time in the presence of hydrogen peroxide, reaching a 50% reduction at the 20-minute mark [66]. The discrepancy between GroEL protein expression and transcript levels may be due to the gradual decrease in transcript levels over 20 min. In contrast, protein expression did not mirror this change, as the samples were collected five minutes after the stress factor application. It is plausible that changes at the proteome level might have been observed if the sampling period was extended. In contrast, *dnaK* expression was stimulated in response to oxidative stress, resulting in a 3-fold upregulation [66]. The abundance of GroEL and GroES proteins remained constant when exposed to an acidic environment [69]. In addition, Mantis [68] demonstrated that GroEL and DnaK protein levels remain unaltered in acidic (pH 5.0) and alkaline (pH 8.7) environments. However, mild acid stress (pH 5.5) has been observed to increase the expression of two genes homologous to *ibpA,* which encode Atu5052 and Atu5449 proteins, by approximately 3.5-fold and nearly 2.5-fold, respectively [70]. Furthermore, CdCl₂ (27 μM) and the antibiotic mitomycin C (10 μg/mL) induced moderate upregulation of DnaK and GroEL proteins. Ultimately, the chaperone proteins DnaK and GroEL contributed to the stress response in the presence of 4% ethanol, which is consistent with the transcriptomic data presented above [68]. The antimicrobial agents t-CNMA and 4-nitro CNMA (cinnamaldehyde derivatives), which are plant-derived bioactive compounds with antiagrobacterial activity, induced a reduction in the expression of *dnaK* and *clpB* genes. After an 8-hour exposure to cinnamaldehyde derivatives, the most significant reduction was observed for 4-nitro CNMA, with a 28-fold decrease in the expression of the *dnaK* gene and a 2.7-fold decrease in *clpB* gene expression. T-CNMA reduced *clpB* expression by approximately 5-fold, whereas no statistically significant decrease was observed for *dnaK expression*. This decrease in expression may be indicative of the antibacterial properties of CNMA derivatives. The concentration of cinnamaldehyde derivatives used (100 μg/mL) significantly inhibited bacterial growth [71]. At this concentration of bioactive compounds, the inhibition of *Agrobacterium* growth in the presence of 4-nitro CNMA was greater than that with t-CNMA. After 24 h, bacterial survival rates were reduced by approximately 60% and more than 90%, respectively. Consequently, it can be inferred that by the eighth hour, cellular death had commenced. This may explain the substantial decrease in *clpB* and *dnaK* expression observed in the presence of 4-nitro CNMA. Moreover, extended exposure to these compounds, lasting up to 8 h, does not preclude the possibility that initial induction may have occurred. However, the kinetics of gene expression over time do not remain constant. In *E. coli* subjected to prolonged exposure to 42 °C, an initial increase in *dnaK* and *clpB* expression was observed. However, after merely one hour, the expression level was lower than that under non-stress conditions, and its decrease continued over time [72].

In *P. syringae* pv. *syringae,* a 3-fold increase in *dnaK* expression was observed for a temperature shift from 18 °C to 35 °C. Additionally, DnaK protein levels increased by approximately 1.5-fold and 4-fold with shifts from 26 °C to 32 °C and from 26 °C to 38.5 °C, respectively. After 5 h of bacterial growth at 32 °C, the DnaK level was significantly lower than the baseline level (time 0), whereas at 38.5 °C, it was comparable to the baseline level [73].

The DnaJ protein of *P. cichorii* JBC1 confers cellular protection against thermal and oxidative stresses. The *dnaJ* mutant exhibited a 20% reduction in cell growth rate at 40 °C and a 60% reduction at 60 °C compared with the wild-type (WT) strain. Moreover, inactivation of the *dnaJ* gene resulted in increased sensitivity to hydrogen peroxide, with a 20% increase within the H_2_O_2_ concentration range of 1–10% [74].

In *P. syringae* pv. *phaseolicola* NPS3121, bacteria cultured at 18 °C exhibited decreased expression of *clpB* (less than 2-fold), *groEL* (more than 3-fold), *grpE*, and *dnaK* (more than 1.5-fold) compared with those cultured at 28 °C. Notably, at lower temperatures, the bacterium causes more severe disease symptoms (halo blight) in common beans through the increased production of phaseolotoxin and induction of chlorosis [75]. However, the culture was conducted without virulence-stimulating plant-derived compounds; thus, the decrease in gene expression encoding chaperones likely resulted from a reduction in temperature. The expression profiles of these genes at low temperatures in planta and with the addition of virulence-inducing agents in vitro remain to be elucidated.

The culture of *P. syringae* pv. *actinidiae* biovar 6 in HS medium at 18 °C induces the production of the phytotoxin coronatine, a significant determinant of this bacterium’s virulence. In the initial phase of infection, coronatine inhibits host defense responses and facilitates the opening of closed stomata, thereby enabling invasion of host tissues [76]. Phytotoxin synthesis was induced at 18 °C, coinciding with reduced expression of chaperone protein genes compared with 27 °C, where toxin production was negligible. *groEL* and *ibpA* expression levels decreased by less than 3-fold and more than 5-fold, respectively, whereas *clpB* levels were unlikely to be induced under the tested conditions [77]. However, this was presumably not attributable to coronatine production. Reducing the bacterial growth temperature results in slower cellular metabolism; consequently, there is likely to be a diminished requirement for chaperone proteins.

In *X. campestris* pv. *campestris,* genes encoding major chaperone proteins are arranged in the order *hrcA*-*grpE*-*dnaK*-*dnaJ.* A temperature shift from 28 °C to 35 °C resulted in a 1.9-fold increase in *dnaK* and a 2.8-fold increase in *grpE* expression. The presence of 4% ethanol induced *dnaK* and *grpE* gene expressions by 1.5-fold and 2-fold, respectively. It was not possible to remove the *dnaK* gene, which may indicate that it is essential or that alternative techniques should be employed [78]. The *hspA* promoter (a homolog of the sHsp protein) of *X. campestris* pv. *campestris* was not induced under acid, alkaline, H₂O₂ oxidative, and ionic osmotic stress conditions and in the presence of SDS. However, gene and protein expressions were induced at 37 °C, with the peak of transcription occurring at approximately 20 min of exposure [60]. HspA protein levels were also elevated [79]. Disruption of the *hspA* gene did not affect virulence in the cabbage leaf model; however, it increased the temperature sensitivity of *X. campestris* in the presence of 40 mM MgSO_4_ at 37 °C, causing a 10-fold reduction in growth compared with that of the WT strain on solid medium. This finding correlates with transcriptomic and proteomic data [60]. GroES protein levels were increased by heat shock at 42 °C [79].

In *D. solani* IPO2222, osmotic stress (both ionic and non-ionic) increased *dnaJ*, *dnaK*, and *groEL* gene expression; however, NaCl (0.3M) had the most pronounced effect. In the exponential growth phase, bacteria showed an 8-fold induction of *dnaJ*, a 15-fold increase in *dnaK* expression, and a 3-fold increase in *groEL* expression. During the stationary phase, fold changes were smaller: 6.5 and 2 for *dnaJ* and *dnaK*, respectively. Consequently, it can be inferred that the DnaK-DnaJ system may play a more significant role in this stress response than GroEL. Additionally, *dnaJ* was upregulated during exponential growth in response to sucrose-induced stress (0.32M sucrose), showing a 4-fold change. In stationary cells, upregulation was observed to be 9-fold for *dnaK* and 2-fold for *groEL*. The greatest induction of gene expression occurred in response to acid stress in the stationary phase, with fold changes of 42, 105, and 116 for *dnaJ, dnaK*, and *groEL,* respectively. However, in the logarithmic phase, induction exceeded 4-fold for *dnaJ* and *dnaK*. Heat stress at 37 °C during the stationary phase caused a 2-fold increase in gene expression, while in the logarithmic phase, the increases were 9-fold (*dnaJ),* 14-fold (*dnaK*), and 17-fold *(groEL*). At 40 °C, *dnaJ* and *dnaK* showed over 3-fold increases in the stationary phase and 10- and 17-fold increases in the logarithmic phase, respectively. *groEL* induction at 40 °C was less significant than that at 37 °C, with over a 5-fold increase observed in the logarithmic phase. Proteome analysis after shifting from 30 °C to 40 °C in the stationary phase showed a 30–40% induction of the proteins encoded by these genes. This increase was statistically significant. The most notable changes were observed in the levels of IbpA and ClpB proteins, which exhibited more than 14-fold and 2-fold increases, respectively [80]. Finally, oxidative stress induced by 0.25 mM H_2_O_2_ caused a slight decrease in the expression of all tested genes, with a statistically significant reduction in *dnaJ* and *groEL* during the exponential phase by over 2 and 12 times, respectively. In contrast, treatment of bacteria with 0.1 mM H_2_O_2_ caused a slight, non-significant reduction in gene expression [81]. In oxidative stress studies, the *D. solani* proteome exposed to 0.25 mM hydrogen peroxide showed no significant protein level changes during the stationary phase compared with non-stress conditions [82]. However, the culture conditions in this study were microaerobic, not aerobic. Similar patterns were observed for ClpB, GrpE, and HtpG, whereas IbpA levels decreased more than 2-fold. *D. solani* is highly susceptible to hydrogen peroxide-induced oxidative stress. A 0.5 mM concentration results in a 4-log reduction in colony-forming units (CFU) per milliliter, with the sublethal concentration around 0.25 mM [81]. Exposure of *D. dadantii* 3937 to oxidative stress also decreased the expression of *dnaJ*, *dnaK*, *groEL*, and other genes encoding Hsp chaperones. During exponential growth, mRNA levels decreased by over 6-fold for *dnaJ,* nearly 5-fold for *dnaK*, and 4-fold for *groEL*. In the stationary phase, reductions were mostly insignificant, except for *groEL*, which decreased by almost 1.5-fold with 0.1 mM hydrogen peroxide exposure [48]. In comparison, for *E. coli* BW25113, the sublethal concentration is approximately 2.5 mM [83]. At sublethal concentrations, an approximately 24-fold increase in *dnaK* expression and a 7-fold increase in *groEL* expression were observed. At a concentration of 1 mM H_2_O_2_, which is significantly lower than the sublethal concentration for *E. coli*, there was moderate upregulation of the *dnaK* and *groEL* genes in this bacterium [84]. Evidence suggests that in *Dickeya*, heat shock chaperones are not involved in protecting cells from oxidative stress. It is hypothesized that these bacteria have developed alternative defense mechanisms against the deleterious effects of free radicals.

In *D. dadantii* 3937, as observed for *D. solani*, genes encoding chaperones are upregulated under salt stress (0.3 M NaCl) during both exponential and stationary growth phases. During intensive cell division, the mRNA levels of *ibpA* and *ibpB* increased over 25- and 38-fold, respectively. In contrast, *dnaJ*, *dnaK*, *grpE*, and *clpB* increased by 4- to 8-fold, whereas *groEL* and *groES* demonstrated an induction of over 1.5-fold. This suggests a minor role for the latter proteins in adaptation to ionic osmotic stress. In stationary-phase cells, the expression levels of *hsp* genes were slightly lower than or similar to those observed in exponential-phase cells. Unlike in *D. solani*, chaperones do not appear to be involved in the adaptation of the cell to low pH (5.0). Gene expression decreased during both phases of growth. During the exponential phase, the *dnaJ, dnaK, htpG, groES,* and *groEL* levels showed a 2-fold reduction. In addition, *grpE*, *groEL*, and *groES* exhibited a 1.5-fold decrease. In the stationary phase, there was a 2-fold reduction in *dnaJ* expression [48].

A 10-fold increase in the expression of the *clpB*, *groEL*, *groES*, *dnaJ*, *hspA*, *dnaK*, and *grpE* genes in *Xylella* was observed following exposure to heat stress at 40 °C for 25 min. The expression of these genes is dependent on the RpoH transcription factor [85]. However, in *X. fastidiosa*, heat shock proteins do not exhibit a conventional response to high-temperature stress, irrespective of the duration of exposure and temperature level, up to 47 °C. These proteins remain constitutively expressed, likely due to the low values of codon usage bias observed in *Xylella*, particularly evident in the housekeeping protein group, among others. This phenomenon results in the representation of certain codons at a lower frequency, thereby preventing or hindering increased protein expression under stressful conditions. Constitutive expression of stress proteins may compensate for this deficiency [79].

In *X. fastidiosa* J1a12 cultured in nitrogen-deficient medium, a notable decline in the expression of genes encoding heat shock proteins was observed at 8 and 12 h. Specifically, the expression levels decreased as follows: *groEL* and *groES* by approximately 6-fold; *hspA* by 4-fold; *dnaJ* by more than 2-fold; *dnaK* by nearly 6-fold; and *grpE* by more than 6-fold. Transcription of the *groE* operon relies on both RpoH and the HrcA repressor, as demonstrated in *A. tumefaciens* [65,85]. Therefore, the observed decrease in *groEL* and *groES* expression can be partly attributed to an increase of more than 4-fold in *hrcA* repressor levels by the 8-hour mark. However, *rpoH* expression was elevated 2-fold under these conditions, suggesting that the repression of chaperone expression was mediated by an additional factor [86].

The co-culture of *X. fastidiosa* 9a5c with the endophyte *Methylobacterium mesophylicum* SR1.6/6 resulted in a 50% increase in the expression of stress-related genes, including *groES, groEL*, *dnaK,* and *grpE*, compared with the monoculture. This response is hypothesized to represent a defense mechanism. Specifically, *M. mesophylicum* inhibits *Xylella* growth through competition for iron and phosphorus and by secreting hydrolytic enzymes that degrade the *Xylella* cell wall [87].

In *R. solanacearum*, the expression of *dnaK* increases in response to the presence of daphnetin, a hydroxycoumarin with antibacterial properties [88].

In summary, the induction of heat shock proteins in phytopathogenic bacteria depends on multiple stress factors (Table 2). The increased expression levels of both *hsp* genes and Hsp chaperones in response to elevated temperatures have been extensively documented. In addition, the expression of *hsp* genes is upregulated during osmotic stress. This evidence suggests a role for Hsp in the adaptation of plant pathogenic bacteria to elevated temperatures and fluctuating environmental osmolarity. The involvement of Hsp in the bacterial response to oxidative and acid stress appears to be species-specific. Furthermore, Hsp may contribute to cellular protection against certain antimicrobial compounds and antibiotics.

#### 2.1.2. The Expression Levels of *hsp* Genes and Hsp Chaperones Are Altered During the Process of Plant Infection

*E. amylovora* exhibits an increased expression of *grpE* during infection of immature pear, presumably due to oxidative stress in the host tissue [89]. This response is similar to the elevated expression of *grpE* observed in *E. coli* under moderate oxidative stress conditions [84]. Holtaples [90] conducted a comparative proteomic analysis of two strains of *E. amylovora:* high-virulence PFB5 and low-virulence LMG 2024T, during apple rootstock infection. Notable differences in protein profiles were identified among these strains, particularly in those associated with virulence and amylovoran production. Furthermore, discrepancies in protein expression patterns related to stress defense, specifically heat shock proteins and cold shock proteins (Csps), were identified. Csp proteins are nucleic acid-binding proteins that play a significant role in bacterial viability under cold shock conditions and adaptation to low temperatures [91]. The high-virulence strain exhibited higher levels of DnaK, ClpB, GroES, and CspC than the low-virulence strain. However, *cspC* expression was elevated in the low-virulence strain, although this increase was not statistically significant. Additionally, this strain demonstrated increased levels of GrpE, HtpG, CspG, and CspE. The mRNA levels of *cspA*, *cspE*, and *dnaK* were consistent with the proteomic data; however, only *dnaK* yielded statistically significant results. It appears that a more virulent strain is more likely to engage genes and proteins associated with the heat shock stress response within the plant. In contrast, the less virulent strain generally upregulates cold-shock proteins. These cold shock proteins have been shown to elicit the host’s defensive immune response as they may be recognized by the host as pathogen-associated molecular patterns (PAMPs) [92]. This recognition may ultimately contribute to the low-virulence phenotype of this strain to some extent [90]. Subsequently, the protein profiles of these strains were compared under in vitro culture conditions and during infection of apple rootstocks. Under in planta conditions, the low-virulence strain exhibited upregulation of ClpB, HtpG, and CspG, while the high-virulence strain showed elevated levels of DnaK and HtpG compared with bacterial growth in the culture medium. Transcriptomic data supported these observations for CspG (encoded by the *cspA* gene) in a high-virulence strain and for DnaK in a low-virulence strain, with the induction of these genes reaching almost 4-fold in planta [93]. The preference for DnaK induction in the high-virulence strain and CspG induction in the low-virulence strain in planta aligns with the findings of Hottaples [90]. Further research on *A. amylovora* strain 650, characterized by its low virulence, largely corroborates these previous findings. The induction of genes in apple varieties with differing susceptibilities to infection was compared with that observed in in vitro bacterial cultures. The Idared apple variety exhibits susceptibility to infection, whereas the FreeRedstar variety demonstrates resistance, with infection confined to minimal necrotic lesions within the shoots. During the initial phase of infection (24 h) in the highly resistant variety, elevated expression levels were observed for *clpB3*, *dnaJ*, *dnaK*, *grpE*, *htpG*, *ibpA*, and *cspA*. In contrast, during infection of the susceptible variety, there was an increase in the expression of the gene encoding cold shock protein (*cspD*) [94]. This observation aligns with the findings of Hottaples [93], who demonstrated that a strain exhibiting low virulence during infection of a susceptible apple variety generally does not activate primary genes associated with heat shock (*dnaK*). The activation of additional stress-related genes suggests that the environment in the resistant FreeRedstar variety is more stressful for the bacteria [94]. It should be noted that proteomic data indicated the induction of ClpB and HtpG, although a different low-virulence strain and apple variety were used in this study [93]. Furthermore, the stage of infection at which samples were collected differed between the two studies. In the proteome study mentioned above, samples were taken late in the systemic infection (10–14 days post inoculation (dpi)), while in the transcriptomic study, samples were collected early in the infection process (1 dpi). These factors, along with the observation that proteomic data do not always correlate with transcriptome analysis [95], may explain the minor discrepancies observed in the data.

HspL is a significant virulence factor in *A. tumefaciens,* as the absence of this protein has been demonstrated to reduce tumor formation in the potato assay by 20–25% compared with the WT strain. This reduction was attributed to a decrease in VirB/D4-mediated DNA transfer by approximately 30%. HspL plays a role in stabilizing VirB protein levels, which subsequently modulates T4SS [63]. The individual deletion of the remaining genes encoding sHsp did not result in a reduction in virulence. However, in the quadruple deletion mutant, a significant decrease in tumorigenesis was observed, approaching 50%, whereas DNA transfer was reduced by 80%. The overproduction of any of these proteins in the quadruple mutant restored tumorigenesis to levels seen in the WT strain, and DNA transfer was 70-190% greater than in the parental strain. This observation suggests that all sHsp of *A. tumefaciens* are significant for the pathogenesis of this bacterium, with HspL being of particular importance, as it is the most abundant sHsp variant following acetosyringone induction [96]. In *A. tumefaciens* C58 ATCC 33970 interacting with an axenic segment of the tomato root, GroEL is represented in three distinct forms, indicating post-translational modifications (PTMs) during its interaction with plant tissues [97]. PTMs are covalent modifications of amino acids that modulate protein properties and functions [98]. Certain proteins may undergo PTMs to adapt to novel environmental conditions. In GroEL, these modifications may include phosphorylation, acetylation, and citrullination, among others [99,100,101]. PTMs of GroEL in the presence of plant extracts may indicate an important role for this chaperone in the infection process. For instance, GroEL in some bacteria, such as *Bacillus anthracis* and *Mycobacterium smegmatis*, undergoes these modifications, thereby modulating biofilm formation [102].

In *P. cichorii* JBC1, the inactivation of *dnaJ* resulted in a reduction in disease symptoms in tomato leaves and cabbage midribs by approximately 50% compared with the WT strain. The necrotic lesions exhibited a lighter coloration in tomato, and disease symptoms manifested as drier and lighter brown in cabbage compared with typical infection progression. Furthermore, the *dnaJ* mutant was unable to elicit a hypersensitivity (HR) response in non-host tobacco leaves within one day post infection. Inactivation of DnaJ resulted in multiple alterations in the phenotype of *P. cichorii*, which may account for the observed reduction in virulence. First, DnaJ contributes to bacterial cell attachment to plant surfaces. This was demonstrated by the observation that the absence of this protein reduced cell abundance in the leaf disk attachment assay by more than two logs (CFU per millimeter squared) compared with the wild-type (WT) strain. Second, the *dnaJ* mutant demonstrated a greater than 3-fold decrease in swarming motility and a 2.5-fold reduction in biofilm formation. Impairment in biofilm formation is attributed to a reduction in extracellular DNA (eDNA) release, which is an important factor governing biofilm integrity. Third, the absence of a functional *dnaJ* gene diminishes susceptibility to oxidative stress encountered by the bacterium during host infection [74]. DnaJ plays an important role in the virulence of animal and human pathogenic bacteria, such as *Edwardsiella tarda* [103] and *Streptococcus pneumoniae* [104].

Disruption of *htpG* (locus *PsgB076_09885*) in *P. savastanoi* pv. *glycinea* resulted in a reduction in necrotic lesions surrounded by chlorosis on soybean leaves compared with the WT strain. The population of mutant bacteria, expressed as log (CFU/g), was isolated from the infected tissues at 6 dpi. This population was approximately two orders of magnitude lower than that of the control strain. Apart from the reduced growth of the *htpG* mutant in the host, the mechanisms underlying its reduced virulence remain unknown [105]. HtpG is implicated in the virulence of the human and animal pathogens *E. tarda* [106], *Leptospira interorgans* [107], *Salmonella enterica* serovar Typhimurium, and extraintestinal pathogenic *E. coli* [108].

In *X. axonopodis* pv. *citrii*, DnaK levels increased during biofilm formation. Under these conditions, both transcript (approximately 4-fold) and protein (approximately 3-fold) induction have been observed, compared with planktonic cell populations [109]. This observation suggests the potential involvement of this chaperone protein in biofilm production and integrity. Previous studies have demonstrated that the downregulation of DnaK *in Streptococcus mutans*, as well as the absence of a functional protein in *Staphylococcus aureus*, results in impaired biofilm formation [110,111].

In *Xanthomonas albilineans* Xa23, HtpG is one of the components involved in the biosynthesis of albicidin phytotoxin, which is responsible for the chlorotic symptoms of sugarcane leaf scald. Furthermore, HtpG co-localizes with the toxin in the cytoplasmic membrane and is also localized in the cytoplasm, although to a lesser extent [112].

ClpB from *X. campestris* pv. *campestris* has been identified as a potential virulence factor. Bacteria cultured in minimal medium that mimics a foliar apoplastic environment and induces the *hrp* regulon (hypersensitive reaction and pathogenicity) associated with T3SS assembly exhibited varying levels of ClpB expression. Specifically, the expression level of ClpB was 2-fold higher in the more virulent Xcc51 strain than in the less virulent XccY2 strain. The transcription level of *clpB* in Xcc51 aligns with proteomic data, indicating a 5-fold induction of *clpB* expression under plant-mimicking conditions compared with XccY2. Furthermore, the levels of GroEL protein were comparable between these strains, whereas GroES exhibited an approximately 2-fold higher relative abundance in Xcc51 [113].

Interestingly, in *X. oryzae* pv. *oryzae* KACC10331, there was approximately a 2-fold reduction in the expression of *groEL*, *groES*, *htpG*, *grpE*, and *dnaK* when cultured in minimal medium that mimicks in planta conditions with the addition of rice leaf extract. A decrease in *grpE* mRNA levels was observed 15 min after the addition of the plant extract, while reductions in the other transcripts occurred at 30 min. After one hour, expression levels returned to baseline [114], suggesting that the increased level of chaperone proteins is not required under these conditions in this particular pathovar. In contrast, the transcript levels of *grpE* and *dnaJ* were upregulated by *Xanthomonas fragariae* during infection of strawberry leaves by more than 2-fold, whereas the transcript level of *dnaK* increased by approximately 1.5-fold. Conversely, *htpG* expression was downregulated by approximately 1.5-fold compared with that in in vitro cultures [115].

During walnut infection with *Xanthomonas arboricola* pv. *juglandis* 417, GroEL and GroES were identified within the 20 most abundant proteins in the *X. abricola* proteome [116]. In *X. citri* subsp. *citri* 306 cells cultured in a medium that induces pathogenicity, four variants of GroEL were observed in the periplasmic-enriched fraction. This finding indicates that GroEL undergoes post-translational modifications in response to host-mimicking conditions [117]. Such modifications may be important for the infection process, as previously speculated for GroEL in *A. tumefaciens.*

In *D. dadantii* 3937 (formerly *E. chrysanthemi* 3937 and *Dickeya chrysanthemi* 3937), the expression levels of *ibpA* and *ibpB* were induced by approximately 1.7-fold and 3.5-fold, respectively, at the 16th hour of infection of African violet leaves compared with the culture medium [118]. This suggests the potential involvement of sHsp chaperones in the pathogenesis of *Dickeya.*

In *X. fastidiosa* strain 9a5c, GroEL and HspA proteins were identified in mature biofilm bacteria, exhibiting differential expression compared with planktonic cells [119]. Conversely, the gene expression of *clpB* and *grpE* increased by approximately 4-fold and 1.5-fold, respectively, compared with that in the planktonic phase [120]. This observation suggests their potential involvement in adaptation mechanisms specific to biofilm conditions or biofilm production. Small Hsp chaperones are known to play a role in biofilm assembly in *E. coli* and *Mycobacterium ulcerans* [121,122], as well as GroEL in *Cronobacter sakazakii* and *Leptospira interrogans* [123,124]. Furthermore, a comparative analysis of the proteome of two virulent *X. fastidiosa* strains, 9a5c (reference strain) and Fb7 (which exhibits increased planktonic behavior but causes more severe symptoms in tobacco), revealed that GrpE was five times more abundant in the former strain [125]. The bacterial culture was conducted in PW broth for seven days, providing optimal conditions for biofilm formation. The primary distinguishing characteristic of these strains is their capacity to produce robust biofilm, particularly 9a5c. Consequently, elevated GrpE levels in this strain may indicate its involvement in this process, although this chaperone has not been identified in the secretory fraction during biofilm formation [125,126]. Recently, the role of GrpE in biofilm formation was confirmed in *Streptococcus suis* [127].

The expression levels of *hsp* genes may depend on the stage of infection. In *X. fragariae* during strawberry infection at 29 dpi (the late phase of infection with visible disease symptoms), *groEL* exhibited a nearly 3-fold reduction in expression compared with that at 12 dpi (the early phase of infection, prior to the manifestation of disease symptoms). This reduction was observed in the majority of the genes involved in host interactions and virulence. This phenomenon can be attributed to the decreased growth rate of bacteria in the advanced phase of infection, resulting from restricted access to nutrients due to the recognition of bacteria by the host. This subsequently leads to a reduction in photosynthesis in leaves [128]. Furthermore, at 12 dpi, water-soaked lesions had not yet manifested, indicating that the bacteria were still in the preparatory phase of infection, which usually begins at 14 dpi [129].

*R. solanacerum* strains GMI1000 and P597, which are not pathogenic at low temperatures, showed increased levels of ClpB protein when grown in co-culture with tomato plant roots, with approximately 5-fold and 3-fold increases, respectively, at 30 °C compared with the levels observed at 18 °C. Additionally, both strains exhibited a 2-fold increase in GroEL protein at 30 °C relative to 18 °C. Furthermore, GM1000 showed an approximately 50% increase in HtpG abundance, whereas GroES levels exhibited a more than 2-fold increase. In contrast, P597 displayed no temperature-dependent changes in chaperone protein levels when cultured without plant components (30 °C vs. 18 °C). This finding suggests that chaperone synthesis is induced in response to plant compounds. Moreover, the levels of GroES and GroEL proteins were reduced by more than 50% at 18 °C in strains virulent at low temperatures (P673 and UW551) in the presence of tomato roots. In the GMI1000 and P597 strains co-cultured with tomato roots at 30 °C compared with 18 °C, *clpB* gene expression was upregulated 10- and 20-fold, respectively, whereas *htpG* expression was nearly 10 times higher. This observation suggests that these proteins are indirectly involved in the virulence of strains by providing enhanced cellular protection [130]. In strains that exhibit virulence at low temperatures, despite demonstrating greater virulence at 30 °C [131], lower temperatures induced the synthesis of GroES and GroEL proteins. This may suggest that these bacteria experience more significant adaptive stress during infection at lower temperatures than under optimal conditions.

One of the genes found to be upregulated in *R. solanacearum* isolated from the xylem of a susceptible heirloom tomato cultivar is *grpE*, which is classified as being expressed in planta. This indicates that *grpE* is induced during xylem colonization and the development of wilt symptoms [132].

The modulation of Hsp levels during plant infection, under conditions mimicking infection, and during biofilm formation suggests the involvement of chaperones in these processes (Table 2). However, transcriptomic and proteomic data alone are insufficient to determine whether chaperones contribute to adaptation to changing microenvironmental conditions during infection, function as virulence factors, or potentially serve both roles. Furthermore, the modulation of chaperone levels depends on the species, strain, host, and specific infection conditions. Direct involvement in the virulence process of phytopathogenic bacteria has been demonstrated for DnaJ from *P. cichorii*, sHsp from *A. tumefaciens,* and HtpG from *P. savastanoi* pv. *glycinea* strains lacking functional chaperones exhibit reduced disease symptoms in plants.

#### 2.1.3. Heat Shock Proteins Are Identified in Extracellular Milieu or on the Bacterial Surface

Secretome analysis of two *E. amylovora* isolates from raspberry cultivated in *hrp*-inducing medium that mimics in planta conditions revealed the presence of DnaK. This protein was not detected in the two additional isolates derived from pear and apple. Isolates from raspberry cannot infect apple and pear, whereas those from apple and pear can cause disease symptoms in raspberry; therefore, the latter have a wider host range [133]. The methodologies employed were consistent across all isolates. If raspberry isolates exhibit comparable resistance to lysis under experimental conditions as other strains, DnaK secretion may be a distinctive characteristic of *E. amylovora* isolated from raspberries. Furthermore, in the *E. amylovora* 273 strain subjected to *hrp*-inducing conditions, GroEL was identified among the extracellular proteins [134].

In *P. syringae* pv. *Tomato* DC3000, DnaK was identified in the extracellular fraction of proteins when cultured under *hrp*-inducing conditions [135]. In a separate study, this pathovar was cultivated in complete KB medium, where HtpG was detected in outer membrane vesicles (OMVs). However, when vesicles were formed under conditions mimicking the apoplastic environment (minimal medium), this chaperone was not detected [136]. Autolysis of bacteria cultured in a rich medium occurs more readily than that of bacteria cultured in a minimal medium [137]. It is likely that cell lysis occurred in KB medium, as cytoplasmic proteins such as ClpP protease and ribosomal proteins were present in the secretome of bacteria grown under these conditions but absent when cultured in minimal medium. This observation, along with the absence of HtpG in the secretory fraction under infection-mimicking conditions, suggests that this chaperone is unlikely to have extracellular functions related to *P. syringae* virulence.

Carnielli [138] identified DnaK and GroEL on the surface of *X. campestris* pv. *campestris* cells during lime leaf infection. The proteins were observed in multiple forms, with a greater number of forms compared with in vitro culture, indicating the occurrence of PTMs. This observation suggests the involvement of DnaK and GroEL in bacterial–host interactions. Furthermore, Ferreira [139] identified the GroEL protein of *X. citri* subsp. *citri* strain 306 pathotype A (*Xac*) in the secretome, but only under nutrient-rich conditions. Notably, GroEL was not detected in the culture medium mimicking pathogenic conditions, which is inconsistent with the reported characteristics of other phytopathogenic bacteria. Additionally, Carneilli [138] cited unpublished data from Ferreira’s team, indicating that DnaK and GroEL were present in the extracted fraction after three days of *Xac* infection of lime. As these data have not been published, they should be interpreted with caution. The secretion of DnaK and GroEL into the extracellular space during rice leaf infection has also been demonstrated in another *Xanthomonas* species, *X. oryzae* pv. *oryzae* [140]. However, partial bacterial lysis occurred in planta; therefore, these results should be interpreted with caution. Furthermore, GroEL, DnaK, and GroES were identified in the extracellular protein fraction of *X. oryzae* pv. *oryzae*, both in liquid medium and during rice leaf infection. GroES and DnaK were more abundant in the in vitro secretome; thus, it seems unlikely that their potential presence outside the cell is related to the virulence process. Additionally, GroEL was represented by four distinct forms under all conditions, indicating the occurrence of post-translational modifications. However, two of these forms were present in greater amounts in the secretome under in vitro conditions, while the other two were more abundant in planta, suggesting a potential role for GroEL in adaptation to host conditions [141].

Secretome analysis of *D. solani* IPO2222 identified 573 proteins, including GroEL, DnaK, GrpE, ClpB, and HtpG [82]. However, it was evident that bacterial lysis occurred during the preparation process, as indicated by the high number of proteins found in the secretome. Furthermore, a significant proportion of the identified proteins were of cytoplasmic origin, including as many as 45 ribosomal proteins, which represent the most abundant groups of cytoplasmic proteins. Consequently, the presence of chaperone proteins in the secretory fraction is likely to be incidental. Additionally, GroEL was identified in the extracellular fraction of *D. dadantii* 3937 cultured in LB medium; however, it was absent from minimal medium that induces pectinase production with the addition of chrysanthemum leaf extract and galacturonate for pectinase production stimulation. The high probability of contamination of the preparation due to lysis and the absence of GroEL in extracellular proteins under conditions mimicking infection [142] supports the hypothesis that GroEL may not function as a secretory protein in *D. dadantii* 3937.

In *Pectobacterium zantedeschiae* 9M, GroEL was identified in membrane vesicles (MVs) when bacteria were cultured under two distinct conditions: minimal medium with polygalacturonate (PGA) and minimal medium with potato extract. However, during growth in minimal medium with PGA, numerous cytoplasmic proteins were detected, including as many as 27 ribosomal proteins. It is hypothesized that the outer–inner membrane vesicles (O-IMVs) may have contaminated the MV fraction during preparation [143]. In contrast, only 19 ribosomal proteins were present in the medium containing potato extract, and their abundance was significantly lower than that of ribosomal proteins found in the minimal medium with PGA. Additionally, the number of peptides identified for GroEL via mass spectrometry (MS) was several times higher than that for each ribosomal protein under these conditions. Consequently, the possibility that GroEL is secreted in the presence of plant compounds cannot be excluded. Moreover, in *P. atrosepticum* SCRI1043, GroEL was identified in the secretory fraction of cultures grown in minimal medium supplemented with potato tuber extract [144].

The *Xylella* secretome contains the chaperones DnaK and GroEL. DnaK was present in OMVs during the later stages of biofilm formation by strain 9a5c, which is known to induce disease symptoms in citrus plants. In contrast, strain J1a12, which is unable to develop citrus variegated chlorosis (CVC) symptoms in citrus and does not form a robust biofilm, secreted DnaK only in the presence of calcium ions. GroEL was identified in the OMVs of both strains at different stages of biofilm formation. Notably, calcium stimulation induced GroEL secretion, similar to DnaK, but this occurred exclusively in the non-virulent strain [126]. Calcium has been shown to enhance surface adhesion, biofilm formation, and twitching motility in *Xylella* [145]. The presence of calcium reduces the quantity of secreted proteins while enhancing biofilm formation and ultimately diminishing virulence. Although biofilm formation is undoubtedly a crucial aspect of this bacterium’s pathogenicity, bacterial movement in the planktonic phase within the host appears to be more significant for the development of systemic disease symptoms [125,146]. The secretion of DnaK and GroEL proteins into the extracellular space during biofilm growth has recently been demonstrated in *Acinetobacter baumannii* [147]. Additionally, GroEL and GroES were among the six proteins identified in *Xylella*-infected grapevine leaves, in contrast to their absence from the secretome under in vitro culture conditions [148]. Furthermore, GroEL and DnaK were identified in the *X. fastidiosa* secretome in both monoculture and co-culture with the endophyte *M. mesophylicum* SR1.6/6; notably, they ranked higher among the most abundant secreted proteins during co-culture [87].

**Figure 3:**
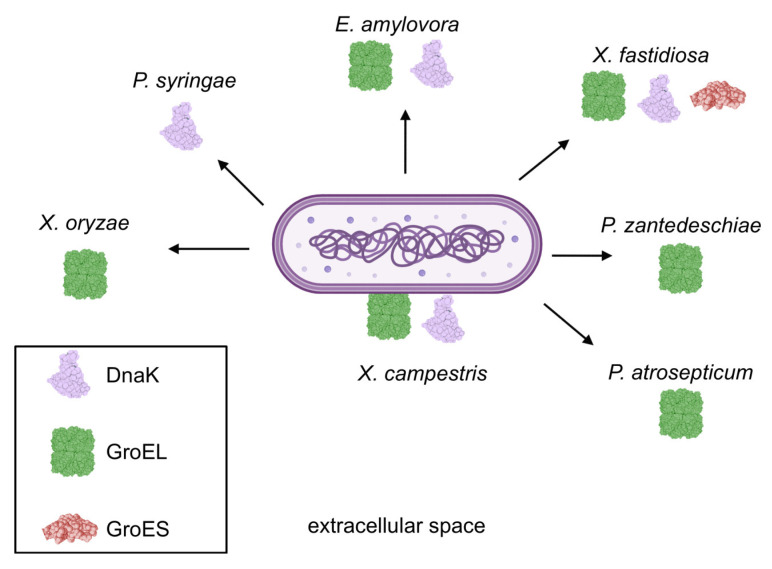
Secretion and surface exposure of Hsp chaperones in phytopathogenic bacteria: This schematic illustrates the bacterial species likely to secrete or expose Hsp proteins outside the cell during plant infection or under conditions that mimic the infection process. This figure does not account for studies in which cell lysis may have occurred.

Currently, there is no evidence that GroEL and DnaK proteins are involved in bacterial–plant host interactions; thus, they exhibit moonlighting functions (i.e., at least two physiologically distinct functions). The moonlighting function of Hsp has been documented in human and animal pathogens. Intracellularly, these proteins act as chaperones to ensure correct substrate folding under both physiological and stressful conditions. Extracellularly, they perform secondary functions related to interactions with the host cells. Outside the cell, GroEL may function as an adhesin by binding to host cells and acting as an intercellular signaling molecule that promotes pro-inflammatory cytokine production by host cells. Not all GroEL homologs exhibit the same moonlighting function [149]. Additionally, among bacteria belonging to the Mollicutes class, certain GroEL homologs are hypothesized to function primarily as adhesins rather than chaperones [150]. In addition to its chaperone role, DnaK binds to plasminogen [151]. Data obtained from secretome analysis (Figure 3) strongly suggest that GroEL and DnaK may be involved in bacteria-plant interactions. The data presented in some of the aforementioned studies indicate that bacterial lysis and the release of cytoplasmic proteins into the extracellular milieu occurred with a high probability. Consequently, these studies were not included in the subsequent analyses. It should be noted that this does not imply a definitive conclusion regarding the absence of chaperones secretion; rather, contamination of the cytoplasmic fraction affects the reliability of their export from the cell. After excluding these data, it appears that (1) the extracellular presence of chaperones is characteristic of virulence-inducing conditions either in vitro or directly in planta, except for one study on *X. citri;* (2) GroEL can undergo post-translational modifications. As previously described, different ratios of GroEL forms are characteristic of cultures under non-inducing infection conditions in vitro and during plant infections, suggesting adaptation for different functions; and (3) the potential for secretion of these proteins appears dependent on both strain and isolate, which may manifest differently depending on bacterial pathogenicity. This suggests that secretion outside the cell is highly probable and may play a role in interactions with host cells. The secretion of GroEL and DnaK has been well documented in both human and animal pathogens. While their roles in host interactions are less well defined, current data suggest that they may modulate host immune responses and facilitate adhesion to host cells. For instance, in *C. sakazakii*, GroEL plays a significant role in adhesion to human enterocyte-like epithelial cells and in the induction of an inflammatory response due to its presence on bacterial surfaces as well as its secretion into the extracellular milieu [123]. GroEL from the animal pathogen *Leptospira* spp. is localized both at the cell surface and within the secretome and has been shown to induce pro-inflammatory cytokine synthesis in macrophages in vitro [152]. In contrast, the probiotic microorganism *Bacillus subtilis* natto secretes GroEL during sporulation and activates both pro-inflammatory and anti-inflammatory cytokines in dendritic cells [153]. In *Mycoplasma pneumoniae*, both DnaK and GroEL are displayed on the cell surface. Recombinant chaperones demonstrated their ability to bind to human cells in vitro, suggesting their role in pathogen adhesion during infection [154]. DnaK from *Mycoplasma hyorhinis* localizes to cell surfaces. It adheres to swine cells and interacts with extracellular matrix components in vitro [155]. Conversely, DnaK from the *Mycoplasma fermentans* PG18 strain has been shown to impair p53-dependent anticancer functions, suggesting potential roles in tumorigenesis [156]. Despite the long-standing recognition that GroEL and DnaK can be exposed outside or secreted by human/animal pathogens, their roles within intracellular environments remain subjects of ongoing investigation. To date, no studies have addressed the interactions between bacterial chaperones belonging to Hsp family and plant proteins; however, such research would yield valuable insights into chaperone functions in phytopathogenic bacteria.

## 3. Conclusions

In comparison with the extensive literature on the role of heat shock proteins in human and animal pathogens, research on these chaperones in phytopathogenic bacteria is relatively limited. To date, only a few investigations have examined the effects of deleting the gene encoding selected heat shock proteins in phytopathogenic bacteria. These studies have focused on their virulence and susceptibility to stress factors. Nevertheless, existing studies provide compelling evidence for the crucial role of these proteins in these processes. In vitro data have clearly demonstrated the involvement of Hsp chaperones in defense against the effects of stressors and adaptation to unfavorable environmental conditions. Less definitive conclusions can be drawn from in planta studies or those conducted in the presence of plant extracts. In this context, the current state of knowledge does not allow for a clear distinction between the protective effects of chaperones and their potential direct involvement in infection as virulence factors or regulators of virulence traits. However, the secretion of certain proteins outside the cell under infection-inducing conditions may suggest a direct role in virulence. This observation may indicate involvement in host interactions, similar to what is observed in bacteria pathogenic to humans and animals.

Future research on heat shock proteins in phytopathogenic bacteria should concentrate on several critical areas. First, constructing additional mutant strains that lack functional Hsp proteins is essential. This will enable the investigation of the effects of these proteins on cellular biology, particularly regarding survival and virulence. If Hsp chaperones are confirmed to play a role in virulence, further examination of the associated impaired virulence traits will be necessary. Second, it is vital to characterize the mechanisms of action through biochemical studies. This includes substrate identification, ATPase activity analysis, and the investigation of oligomerization among selected chaperones. Third, for Hsp proteins with potential extracellular localization, it is crucial to first determine this localization and examine the secretion pathway. After confirming the roles of these extracellular chaperones in adhesion to host cells and their modulation of the host immune response, priority should be given to investigating inhibitors or substances that can (1) block the secretion of Hsp; (2) disrupt the ATPase activity of GroEL and DnaK; and (3) interfere with the oligomerization of GroEL or its interaction with GroES. Additionally, exploring genetically modified plant varieties that, for example, are resistant to the actions of extracellular bacterial GroEL, may offer novel strategies to reduce infection efficiency.

## Figures and Tables

**Table 1:** General characteristics of the selected phytopathogenic bacteria.

Genus	Species Examples	Major Hosts	Disease Symptoms	Example Sets of Virulence Traits	References
*Xanthomonas*	*X. oryzae* *X. campestris* *X. axonopodis* *X. citri*	Rice, citrus, cabbage, broadleaf, carpetgrass	Bacterial blight, citrus canker, black rot	Ax21 protein, motility, biofilm formation, exopolysaccharides (EPS)- xanthan, type III secretion system (T3SS), plant cell wall degrading enzymes (PCWDEs)	[2,36,37,38]
*Erwinia*	*E. amylovora*	Apple, pear	Fire blight	EPS (amylovoran, levan), motility, biofilm formation, T3SS, catalase activity, PrtA protease, siderophores	[2,39,40,41,42]
*Pseudomonas*	*P. syringae* *P. cichorii* *P. savastanoi*	Tomato, tobacco, soy, olive, chicory	Bacterial leaf spot, bacterial blight, plant canker (tumor)	T3SS, biofilm formation, siderophores, motility, EPS (alginate, levan), PCWDEs, coronatine toxin	[2,43,44,45]
*Xylella*	*X. fastidiosa*	Coffee, grapevine, olive tree, citrus	Pierce’s diseases, citrus variegated chlorosis (CVC), coffee leaf scorch	Type II secretion system (T2SS), biofilm formation, afimbrial haemagglutinin adhesins, EPS, type IV-pili-based motility	[2,46,47]
*Dickeya*	*D. dadantii* *D. solani*	Potato, cabbage, chicory	Soft rot, black leg	PCWDEs, chemotactic motility, siderophores	[2,6,12,48]
*Ralstonia*	*R. solanacearum*	Potato, tobacco, peanut	Bacterial wilt	T3SS, EPS, biofilm formation, motility, PCWDEs	[2,49,50]
*Agrobacterium*	*A. tumefaciens*	Grapevine, plum, peach	Crown gall, hairy root	Transferred DNA (T-DNA) transfer via type IV secretion system (T4SS) chemotactic motility, Vir proteins	[2,51,52]
*Pectobacterium*	*P. atrosepticum* *P. carotovorum*	Potato, carrot, tomato, celery	Black leg, soft rot, aerial stem rot	PCWDEs, T3SS, siderophores, motility	[2,53,54,55,56]

**Table 2:** Induction of *hsp* (heat shock protein) genes and Hsp proteins under discussed conditions.

Genus	Hsp (Eat Shock Protein)	Elevated Temperature	Ethanol	Acidic pH	Oxidative Stress	Ionic Osmotic Stress	Non-Ionic Osmotic Stress	Antibiotics	Heavy Metals	Antimicrobial Compounds	Co-Culture with Other Bacteria	In Planta	Mimicking In Planta Conditions	Biofilm Formation
*Agrobacterium*	GroEL	+	+					+	+					
	GroES	+	+											
	DnaK	+	+		+			+	+					
	DnaJ													
	GrpE													
	HtpG													
	ClpB	+												
	Small Hsp	+	+	+										
*Pseudomonas*	GroEL	+												
	GroES													
	DnaK	+												
	DnaJ	+			+									
	GrpE	+												
	HtpG													
	ClpB	+												
	Small Hsp	+												
*Xanthomonas*	GroEL													
	GroES	+												
	DnaK	+										+		+
	DnaJ											+		
	GrpE	+										+		
	HtpG													
	ClpB													
	Small Hsp	+												
*Dickeya*	GroEL	+		+		+	+							
	GroES					+								
	DnaK	+		+		+	+							
	DnaJ	+		+		+	+							
	GrpE					+								
	HtpG													
	ClpB	+				+								
	Small Hsp	+				+						+		
*Xylella*	GroEL	+									+			+
	GroES	+									+			
	DnaK	+									+			
	DnaJ	+												
	GrpE	+									+			+
	HtpG													
	ClpB	+												+
	Small Hsp	+												+
*Ralstonia*	GroEL												+	
	GroES												+	
	DnaK									+				
	DnaJ													
	GrpE											+		
	HtpG												+	
	ClpB												+	
	Small Hsp													
*Erwinia*	GroEL													
	GroES													
	DnaK											+		
	DnaJ											+		
	GrpE											+		
	HtpG											+		
	ClpB											+		
	Small Hsp											+		

+ stands for confirmed upregulation of gene or protein expression.

## Data Availability

Not applicable.

## Abbreviations

ADP	adenosine diphosphate
ATP	adenosine triphosphate
CFU	colony forming unit
Csp	cold shock protein
CVC	citrus variegated chlorosis
dpi	day post infection
eDNA	Extracellular DNA
EPS	exopolysaccharides
HR	hypersensitive response
*hrp*	hypersensitive reaction and pathogenicity genes
Hsp	heat shock protein
sHsp	small heat shock protein
MS	mass spectrometry
MV	membrane vesicle
O-IMV	outer–inner membrane vesicles
OMV	outer membrane vesicle
PAMP	pathogen-associated molecular pattern
PTM	post-translational modification
SBD	substrate-binding domain
T-DNA	transferred DNA
T2SS	type II secretion system
T3SS	type III secretion system
T4SS	type IV secretion system
T6SS	type VI secretion system
WT	wild-type
